# Ancient Plant Glyoxylate/Succinic Semialdehyde Reductases: GLYR1s Are Cytosolic, Whereas GLYR2s Are Localized to Both Mitochondria and Plastids

**DOI:** 10.3389/fpls.2017.00601

**Published:** 2017-04-21

**Authors:** Carolyne J. Brikis, Adel Zarei, Christopher P. Trobacher, Jennifer R. DeEll, Kazuhito Akama, Robert T. Mullen, Gale G. Bozzo, Barry J. Shelp

**Affiliations:** ^1^Department of Plant Agriculture, University of Guelph, GuelphON, Canada; ^2^Ontario Ministry of Agriculture Food and Rural Affairs, SimcoeON, Canada; ^3^Department of Biological Science, Shimane UniversityMatsue, Japan; ^4^Department of Molecular and Cellular Biology, University of Guelph, GuelphON, Canada

**Keywords:** abiotic stress, γ-aminobutyrate, glyoxylate/succinic semialdehyde reductase, photorespiration, phylogenetic analysis, subcellular localization

## Abstract

Plant NADPH-dependent glyoxylate/succinic semialdehyde reductases 1 and 2 (GLYR1 and GLYR2) are considered to be involved in detoxifying harmful aldehydes, thereby preserving plant health during exposure to various abiotic stresses. Phylogenetic analysis revealed that the two GLYR isoforms appeared in the plant lineage prior to the divergence of the Chlorophyta and Streptophyta, which occurred approximately 750 million years ago. Green fluorescent protein fusions of apple (*Malus* x *domestica* Borkh.), rice (*Oryza sativa* L.) and *Arabidopsis thaliana* [L.] Heynh GLYRs were transiently expressed in tobacco (*Nicotiana tabaccum* L.) suspension cells or Arabidopsis protoplasts, as well in methoxyfenozide-induced, stably transformed Arabidopsis seedlings. The localization of apple GLYR1 confirmed that this isoform is cytosolic, whereas apple, rice and Arabidopsis GLYR2s were localized to both mitochondria and plastids. These findings highlight the potential involvement of GLYRs within distinct compartments of the plant cell.

## Introduction

Plant NADPH-dependent glyoxylate/succinic semialdehyde (SSA) reductases (GLYR, EC 1.2.1.79) are hypothesized to detoxify harmful reactive aldehydes into their corresponding less toxic alcohols, thereby preserving plant health during various abiotic stresses (Allan et al., 2009). Glyoxylate is an intermediate in photorespiration and produced in peroxisomes via the oxidation of glycolate (Bauwe et al., 2010), whereas SSA is an intermediate in γ-aminobutyrate (GABA) metabolism and produced in mitochondria via the transamination of GABA (Shelp et al., 2012).

GLYRs were first isolated from spinach and pea leaves, and found in both cytosolic and purified plastid fractions, with 10–20% of the total leaf GLYR activity being present in isolated chloroplasts (Zelitch, 1953; Givan et al., 1988; also see review by Givan and Kleczkowski, 1992). To date, the best characterized plant GLYRs in terms of their subcellular localizations and biochemical properties are those from Arabidopsis. For instance, *At*GLYR1 has been shown to be localized to cytosol, whereas *At*GLYR2 is localized to plastids (Simpson et al., 2008; Ching et al., 2012). Both enzymes prefer NADPH over NADH as a cofactor and display a higher affinity for glyoxylate than for SSA (Breitkreuz et al., 2003; Hoover et al., 2007a; Simpson et al., 2008), and NADP^+^ competitively inhibits *At*GLYR1, indicating that NADPH/NADP^+^ ratios may regulate GLYR activity *in planta* (Hoover et al., 2007b). In the current study, we studied evolutionary relationships between the two plant GLYRs and compared the subcellular localization of GLYRs from apple (*Malus* × *domestica* Borkh.), a dicotyledonous species, and rice (*Oryza sativa* L.), a monocotyledonous species, with those from *Arabidopsis thaliana* [L] Heynh. Our findings established that GLYR1s are exclusively cytosolic, whereas GLYR2s are localized to both mitochondria and plastids.

## Materials and Methods

### Phylogenetic Analysis

Arabidopsis GLYR1 and GLYR2 proteins were used as queries for a BLASTP search of the National Center for Biotechnology Information^1^, Phytozome^2^, and OneKP^3^ databases. To construct the phylogenetic tree, GLYR proteins were chosen from among chlorophytic and streptophytic species with identity above a 50% cutoff; their NCBI Reference Sequence IDs are given in Supplementary Tables S1, S2. The evolutionary history was inferred using the Maximum Likelihood method based on the JTT matrix-based model (Jones et al., 1992). The tree with the highest log likelihood (–8865.0032) is shown. All positions containing gaps and missing data were eliminated. Evolutionary analysis was conducted in MEGA7 (Kumar et al., 2016).

### Plant Materials, RNA, and DNA Extraction, and Identification of Plant GLYRs

*Arabidopsis thaliana* (L.) Heynh ecotype Columbia (Col-0) was the genetic background of the wild type (WT) and the *AtGLYR2*- *GREEN FLUORESCENT PROTEIN* (*GFP*) transgenic line. Total RNA was extracted and used for synthesis of cDNA and quantitative PCR analysis as described previously (Zarei et al., 2011, 2014). The preparation of apple and rice RNA and cDNA has been described elsewhere (Brauer et al., 2011; Trobacher et al., 2013b). The primer sequences used to determine the abundance of *GLYR2*-*GFP* and the housekeeping transcript *ELONGATION FACTOR-1 ALPHA* (At5g60390; Czechowski et al., 2005) are listed in Supplementary Table S3. The extraction of Arabidopsis genomic DNA has been described (Zarei et al., 2011).

### Identification and Cloning of cDNAs Encoding Apple and Rice GLYRs and Arabidopsis GLYR2

The Arabidopsis *GLYR* sequences were utilized as queries in the apple genome database^4^. Two GLYRs have been identified as *Md*GLYR1 (MDP0000149834) and *Md*GLYR2 (MDP0000158245). GFP-tagged versions of *Md*GLYR2, *At*GLYR2, and *Os*GLYR2 were constructed for subcellular localization studies. The full-length open reading frame (ORF) of *MdGLYR1* was amplified with CB-F1 and CB-R1 primers, whereas the *MdGLYR2* ORF was amplified with CB-F2 and CB-R2 primers (Supplementary Table S3). The resulting PCR products were sub-cloned into the plant expression vector pUC18-GFP, resulting in both *Md*GLYRs being fused at their C-termini to a monomerized version of GFP. The full-length ORF of *AtGLYR2* was amplified with *Nhe*I-AtGLYR2-F and *Nhe*I-AtGLYR2-R primers from Arabidopsis rosette leaf cDNA. The resulting PCR product was sub-cloned into pUC18/*NheI*-GFP, yielding *AtGLYR2*-GFP. The rice cv. Nipponbare (AK064876) cDNA was provided by the Rice Genome Resource Center, National Institute of Agrobiological Sciences (Tsukuba, Japan). The *OsGLYR2* ORF was amplified using the primer sets *Not*I-OsGLYR2-F and *Xho*I-OsGLYR2-R (Supplementary Table S3). The resulting PCR product was sub-cloned into pTH-2 vector (Englert et al., 2007), yielding *OsGLYR2-GFP*.

### Transient Expression and Subcellular Localization of Apple, Rice, and Arabidopsis and Rice GLYRs in Tobacco BY-2 Cells and Arabidopsis Protoplasts

A total of 5 μg of plasmid DNA encoding an individual GFP fusion protein with or without 1 μg of plasmid DNA encoding cytosolic Cherry (pRTL2/Cherry; Gidda et al., 2011) or the plastidial marker pSAT4/PDCpl-E2-Cherry (Park et al., 2012) was transiently expressed in tobacco BY-2 suspension cells via tungsten particle bombardment. Details on processing BY-2 cells for (immunofluorescence) microscopy, including cell fixation, as well as confocal laser-scanning microscopy (CLSM) are described in Trobacher et al. (2013a). Mitochondria were immunostained using rabbit anti-CoxII affinity-purified IgGs and goat anti-rabbit rhodamine red × secondary antibodies (Jackson Immunoresearch Laboratories), according to Frelin et al. (2012). GFP and chlorophyll [in Arabidopsis protoplasts or seedlings (see below)] were excited with a 488 nm argon ion laser at 25% power, and the emitted light was detected at 500–530 nm for GFP and 685–750 for chlorophyll. Cherry and Mitotracker were excited with a 543 nm Ar/HeNe laser at 85% power, and emitted light was detected at 590–650 nm for Cherry and 579–599 nm for Mitotracker.

Arabidopsis cell suspension protoplasts were prepared by enzyme digestion (Zarei et al., 2011). Ten microgram of plasmid DNA encoding an individual GFP fusion protein with or without 5 μg of pSAT4/PDCpl-E2-Cherry plasmid DNA was mixed with 125 μL of protoplast solution containing half a million cells. Protoplasts were transformed using polyethylene glycol as described elsewhere (Schirawski et al., 2000). Mitochondria were stained with Mitotracker Red CMXRos (Thermo Fisher Scientific) as described in the manufacturer’s manual.

All fluorescent images of BY-2 cells and protoplasts are representative of at least three independent transformations with a minimum of 10 transformed cells imaged per transformation. Fluorophore emissions were imaged sequentially, and no detectable bleed-through was observed with the same acquisition settings used in data collection.

### Stable Expression and Subcellular Localization of *At*GLYR2 in Arabidopsis Seedlings

A stable Arabidopsis line expressing methoxyfenozide-inducible *AtGLYR2*-*GFP* was generated as described by Dietrich et al. (2008). The *AtGLYR2*-*GFP* ORF was amplified from pUC18-*AtGLYR2-GFP* with *F-PacI-LAtGR2-GFP* and *R-SpeI-LAtGR2-GFP* primers and sub-cloned into the plasmid CD1660-1-5XG-M35S, resulting in the construct CD-1660-1-5XG-M35S::*AtGLYR2*-*GFP*. The 5XG-M35S-*AtGLYR2*-GFP cassette was digested with *Not*I and *Apa*I and subcloned into the plasmid CD1468-1 possessing a promoter-binding VGE element, resulting in the construct CD1468-1-VGE-5XG-M35S::*AtGLYR2*-*GFP*. This cassette was then digested with *Asc*I and sub-cloned into the binary vector pEC291and transformed into EHA105 *Agrobacterium* cells. Arabidopsis plants were stably transformed with pEC291-*AtGLYR2-*GFP via the floral dip method (Clough and Bent, 1998) and PCR-positive transgenic plants were further tested for protein expression and phenotype. Highly expressing GFP lines of 14-days-old T_2_ seedlings were selected using an epifluorescent microscope (Leica DM-6000CS), then grown to maturity for collection of T_3_ seed.

Localization analysis of Arabidopsis stably expressing inducible-*AtGLYR2*-GFP was performed using 14-days-old T_3_ seedlings. Expression of *AtGLYR2-*GFP was induced by foliar application of 61 μM methoxyfenozide (Intrepid 2F insecticide, Dow AgroSciences). Seedlings were incubated for 48 h before imaging. Microscopy was performed using CLSM as described above.

## Results and Discussion

### Comparative Genomic Analysis of Plant GLYRs

**FIGURE 1:**
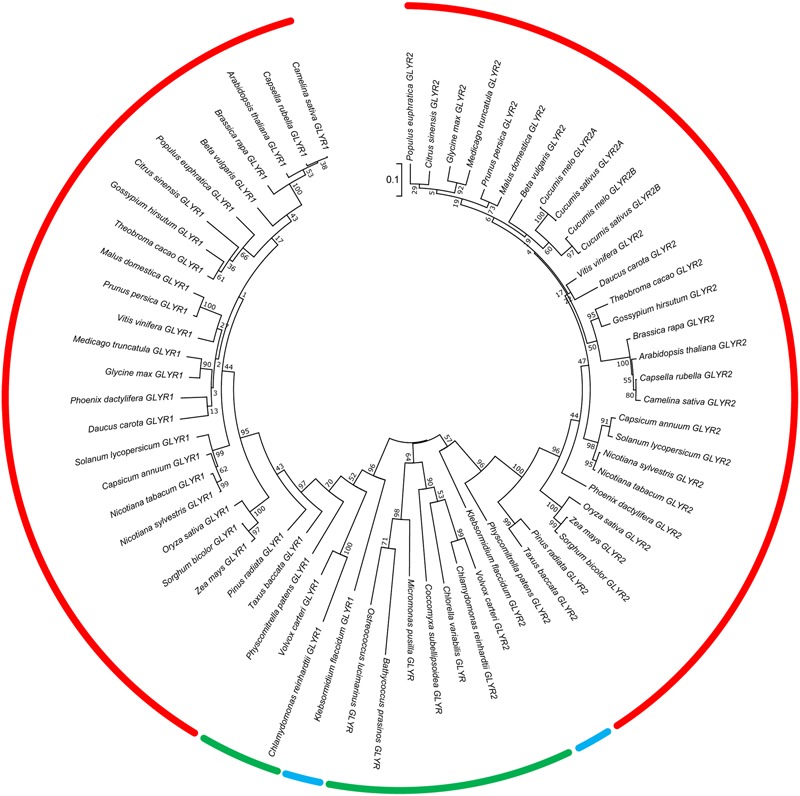
**Phylogenetic analysis of plant GLYR amino acid sequences across plant species.** The unrooted phylogenetic tree was depicted using the Maximum Likelihood method in MEGA7. The red, blue, and green bars represent species in the Embryophyta, Charophyta, and Chlorophyta, respectively. The numbers at each node represent support values obtained by a bootstrap test using 1000 replicates (Felsenstein, 1985). The tree is drawn to scale (0.1 representing a 10% change) with branch lengths in the same units as those of the evolutionary distances used to infer the phylogenetic tree. The protein IDs for the GLYR1s and GLYR2s are given in Supplementary Tables S1, S2.

The deduced amino acid sequences of GLYR1 and GLYR2 have 54–58% identity across 35 species of Viridiplantae, which is comprised of chlorophytic species, including both unicellular and multicellular algae, as well as charophytic and embryophytic plants (the latter two groups jointly known as the Streptophyta) (Becker, 2013). These sequences were used to construct a phylogenetic tree (**Figure 1**). The GLYR1 and GLYR2 proteins from the Embryophyta generated two distinct clusters with a relatively high degree of sequence identity within each cluster (78–97% for GLYR1s and 66–84% for GLYR2s). A small clade with a high degree of identity (85–91% for GLYR1 and 73–85% for GLYR2) is dedicated to monocotyledonous plants within each cluster. Most of the embryophytic genomes contain both *GLYR1* and *GLYR2* genes, the notable exceptions being *Cucumis sativus* and *C. melo*, which lack *GLYR1*, but apparently have two *GLYR2*s located in tandem on the same chromosome. The two GLYR2s in *C. sativus* and *C. melo*, which were designated as GLYR2A and GLYR2B, are 72–73% identical. Furthermore, *in silico* analysis of subcellular localization using TargetP (Emanuelsson et al., 2000) and WoLF PSORT (Horton et al., 2007) revealed that these cucurbit GLYR2As, like their Arabidopsis, apple and rice counterparts (Supplementary Figure S1A), possess a putative N-terminal mitochondrial/chloroplastidial targeting sequence, whereas the cucurbit GLYR2Bs do not. *Physcomitrella patens, Klebsormidium flaccidum, Chlamydomonas reinhardtii*, and *Volvox carteri* contain both GLYR1 and GLYR2 proteins. However, other members of the Chlorophyta (*Chlorella variabilis, Coccomyxa subellipsoidea* C-169, *Micromonas pusila, Bathycoccus prasinos*, and *Ostreococcus lucimarinus*) contain only a single GLYR, with 46–53% identity to *At*GLYR1.

Overall, we found that both GLYR1 and GLYR2 appear in the plant lineage prior to the divergence of the Chlorophyta and Streptophyta, which occurred approximately 750 million years ago (Becker, 2013). Notably, only a single GLYR is found in the oldest members of the Chlorophyta examined and the number of GLYR proteins has not expanded during evolution of the Streptophyta. Thus, the GLYR1/2 orthologs probably arose from the duplication of a single GLYR-type protein in the Chlorophyta, which underwent functional diversification. Also, an extinction event, followed by duplication, probably occurred after the divergence of the Cucurbitaceae, resulting in subfunctionalization of the GLYR2 proteins (Brocker et al., 2013). Hoover et al. (2013) have identified six important amino acid residues for catalysis (Lys170 and Asn174 in *At*GLYR1) and substrate binding (Phe231, Asp239, Ser121, Thr95 in *At*GLYR1) and protein sequence alignment of the GLYRs studied here revealed a general conservation of these catalytic and substrate binding residues, with only two exceptions. *C. reinhardtii* GLYR1 lacks both catalytic residues (Lys170 and As174), whereas *C. variabilis* GLYR1 lacks Thr95, suggesting that the majority of the GLYRs investigated here are indeed functional. Interestingly, single GLYRs also appear in the bacterial lineage (e.g., *Geobacter spp;* Zhang et al., 2014), and these possess greater identity to the plant GLYR1s than the GLYR2s (approximately 50–52%). Based on conservation of all the plant catalytic and substrate binding residues, it seems likely that these GLYRs would also prefer glyoxylate over SSA as substrate, but further study is required to substantiate such a prediction.

### Subcellular Localization of Apple, Rice, and Arabidopsis GLYRs in Transient and Stable Expression Plant (cell) Systems

Apple, rice, and Arabidopsis GLYR1s lack a predicted N-terminal organelle targeting signal (Supplementary Figure S1A) and are therefore considered to be cytosolic. Indeed, while *At*GLYR1 was previously thought to contain a C-terminal tripeptide sequence (i.e., SRE) similar to the type 1 peroxisomal target signal (PTS1) motif (reviewed in Reumann et al., 2016), recent experimental evidence indicates that the protein is localized exclusively to the cytosol and not to peroxisomes (Ching et al., 2012). Both *Md*GLYR1 and *Os*GLYR1 lack a C-terminal PTS1-like peroxisomal targeting signal and are therefore presumed to be also exclusively cytosolic.

Compared to their GLYR1 counterparts, most higher plant GLYR2s examined to date (cucurbit GLYR2Bs being the exception known) possess N-terminal extensions of varying lengths and are generally predicted to be localized to plastids rather than to mitochondria (or the cytosol) by various subcellular localization prediction programs (Supplementary Figures S1A,B). A mitochondrial-specific prediction program such as MitoProt (Claros, 1995) predicts mitochondrial localization for all three GLYR2s, whereas another, MitoFates (Fukasawa et al., 2015) predicts mitochondrial localization for rice GLYR2 only. However, DualPred, a program specific for dual targeting to mitochondria and plastids (Saravanan and Lakshmi, 2014), predicts that *Md*GLYR2 and *Os*GLYR2, but not *At*GLYR2, localize to both organelles. Moreover, *At*GLYR2 is annotated at the Arabidopsis subcellular proteome database [SUBAcon (Hooper et al., 2014)] to be localized to plastids; however, this latter conclusion was drawn solely from mass spectrometry studies involving isolated plastids (i.e., chloroplast), but not mitochondrial organellar fractions.

**FIGURE 2:**
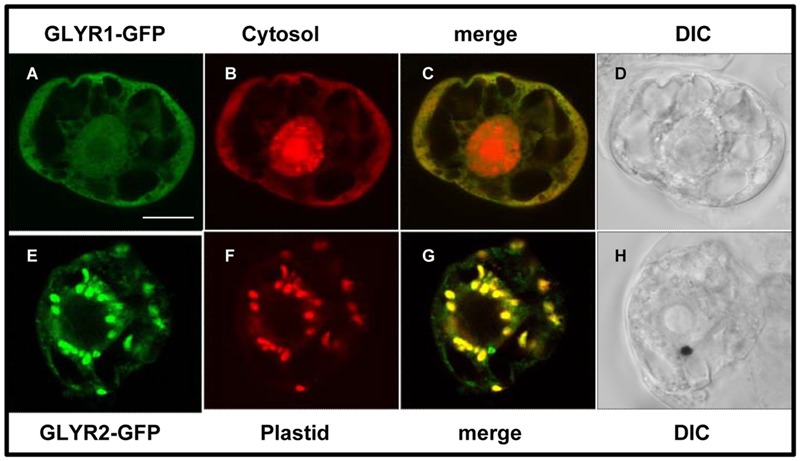
**Subcellular localization of *Md*GLYRs in tobacco BY-2 cells.** Representative CLSM images of BY-2 cells (co)expressing (as indicated by the panel labels) *MdGLYR1-GFP* **(A)** and the cytosolic marker *Cherry* **(B)** or MdGLYR2-GFP **(E)** and plastid marker PDC_pl_-*E2-mCherry* **(F)**. Processing of cells and viewing using CLSM are described in the Section ‘Materials and Methods’. Shown also is the corresponding merged **(C,G)** and differential inference contrast (DIC) **(D,H)** images for each cell. The yellow color in the merged images indicates co-localization. Scale bar in A = 10 μm.

**FIGURE 3:**
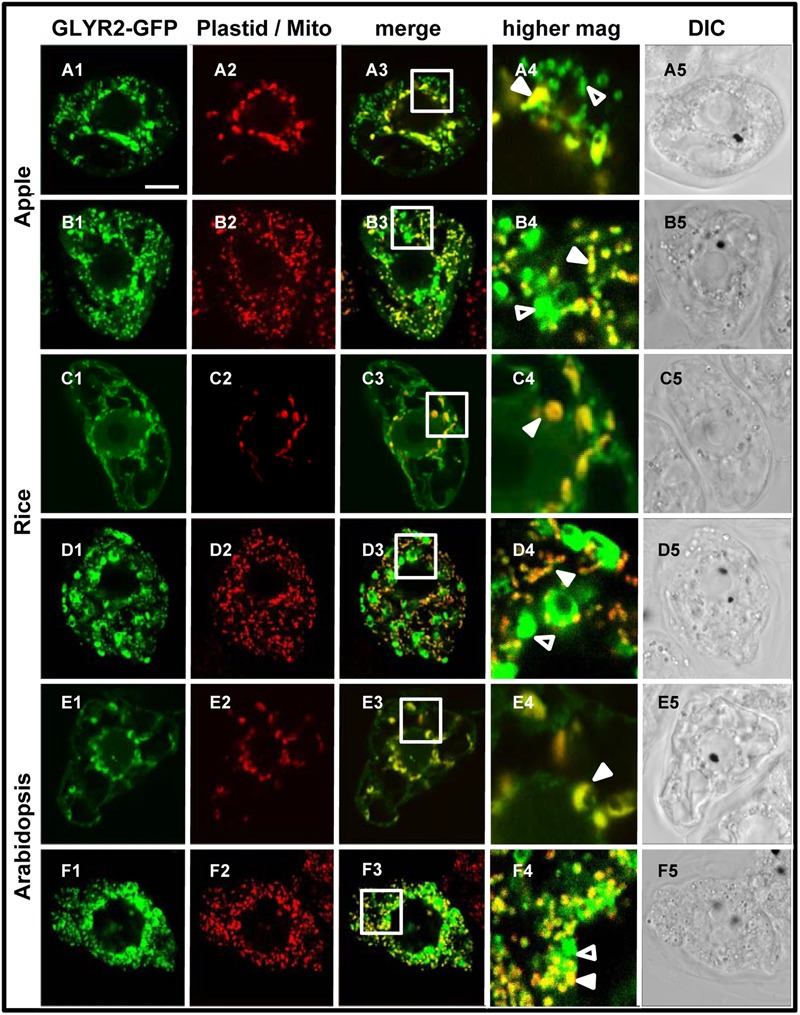
**Plastid and mitochondrial localization of *Md*GLYR2, *At*GLYR2, or *Os*GLYR2 in tobacco BY-2 cells. (A–F)** Representative CLSM images of BY-2 cells (co)expressing (as indicated by the panel labels) *Md*GLYR2-GFP, *Os*GLYR2-GFP, or *At*GLYR2-GFP and either the plastid marker PDC_pl_-E2-Cherry or immunostained for endogenous, mitochondrial CoxII. Processing of cells for immunofluorescence and viewing using CLSM are described in the ‘Materials and Methods’. Shown also is the corresponding merged and DIC images for each cell. Boxes in the merged images represent the portion of the cell shown at higher magnification in the panel to the right. The yellow color in the merged images indicates co-localization; solid arrowheads indicate obvious examples of co-localization, whereas open arrowheads indicate obvious examples of non-co-localization. Scale bar in A = 10 μm.

In order to begin to assess the subcellular localization of GLYRs *in vivo, Md*GLYR-GFP fusion proteins were transiently expressed and visualized via CLSM in tobacco suspension-cultured BY-2 cells, serving as a well-established model system for protein subcellular localization studies (Brandizzi et al., 2003). Similar to the abovementioned previous results for *At*GLYR1 (Ching et al., 2012), *Md*GLYR1-GFP displayed diffuse localization throughout the cell, similar to co-expressed Cherry, a red fluorescent protein that served as a cytosolic marker protein (Shaner et al., 2004) (**Figures 2A–D**). By contrast, *Md*GLYR2-GFP localized to distinct globular-shaped structures, which co-localized with the co-expressed plastid marker fusion protein, consisting of the E2 subunit of the pyruvate dehydrogenase complex fused to Cherry (PDC_pl_-E2-Cherry) (Park et al., 2012) (**Figures 2E–H**), indicating that, similar to previous results for *At*GLYR2 (Simpson et al., 2008), *Md*GLYR2 is localized to plastids. However, *Md*GLYR2-GFP in the majority (>70%) of BY-2 cells examined localized also to numerous small puncta that did not co-localize with co-expressed PDC_pl_-E2-Cherry (**Figure 3A**), but did co-localize with the endogenous mitochondrial protein, cytochrome oxidase subunit II (CoxII) (**Figure 3B**). Taken together, these data suggest that *Md*GLYR2-GFP is localized to both plastids and mitochondria in BY-2 cells. Similar results were observed for *Os*GLYR2-GFP (**Figures 3C,D**). These findings also prompted us to revisit the subcellular localization of *At*GLYR2, which had not been assessed for mitochondrial localization in our previous study (Simpson et al., 2008). As shown in **Figures 3E,F**, *At*GLYR2-GFP localized to both co-expressed PDC_pl_-E2-Cherry-containing plastids, as well as to endogenous CoxII-containing mitochondria.

**FIGURE 4:**
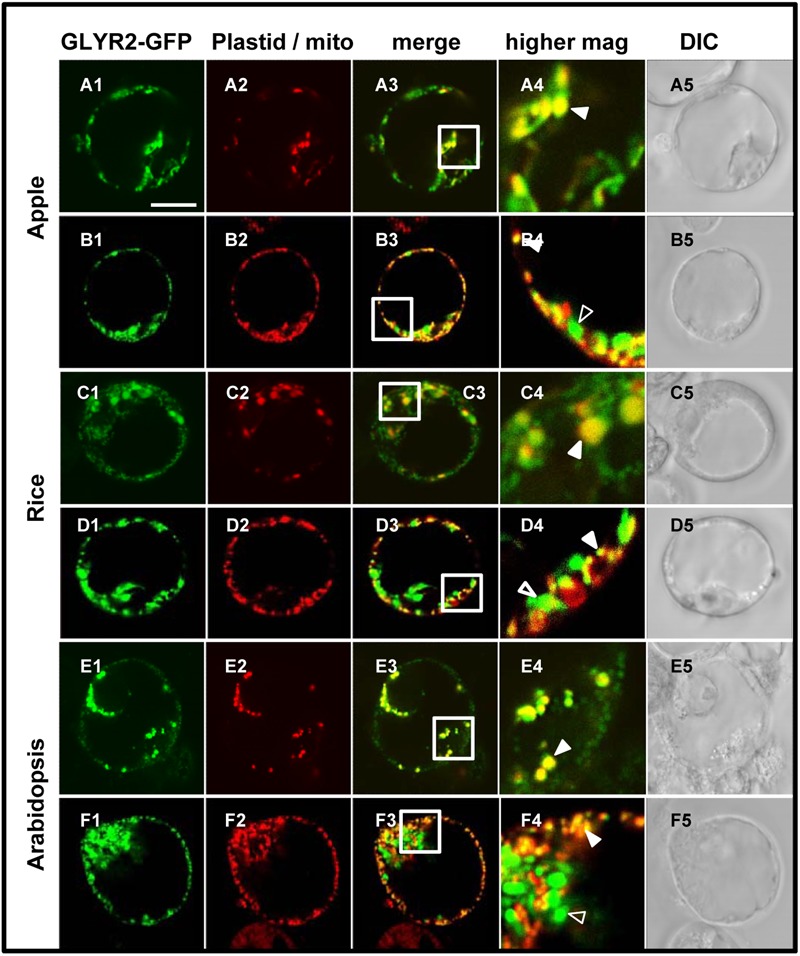
**Plastid and mitochondrial localization of *Md*GLYR2, *Os*GLYR2, or *At*GLYR2 in Arabidopsis protoplasts. (A–F)** Representative CLSM images of Arabidopsis protoplasts (co)expressing (as indicated by the panel labels) *Md*GLYR2-GFP, *Os*GLYR2-GFP, or *At*GLYR2-GFP and either the plastid marker PDC_pl_-E2-Cherry or stained with Mitotracker. Processing and viewing of protoplasts using CLSM are described in the Section ‘Materials and Methods’. Shown also is the corresponding merged and DIC images for each cell. Boxes in the merged images represent the portion of the cell shown at higher magnification in the panel to the right. The yellow color in the merged images indicates co-localization; solid arrowheads indicate obvious examples of co-localization, whereas open arrowheads indicate obvious examples of non-co-localization. Scale bar in A = 10 μm.

In order to assess the possibility that artifacts could be generated by heterologous protein expression in tobacco BY-2 cells (reviewed in Denecke et al., 2012 and Millar et al., 2009), the subcellular localization of *Md*GLYR2-GFP, *Os*GLYR2-GFP, and *At*GLYR2-GFP was further investigated in transiently transformed Arabidopsis suspension cell-derived protoplasts. As shown in **Figure 4**, all three GLYR2-GFPs localized in protoplasts to both co-expressed PDC_pl_-E2-Cherry-containing plastids and Mitotracker-stained mitochondria.

**FIGURE 5:**
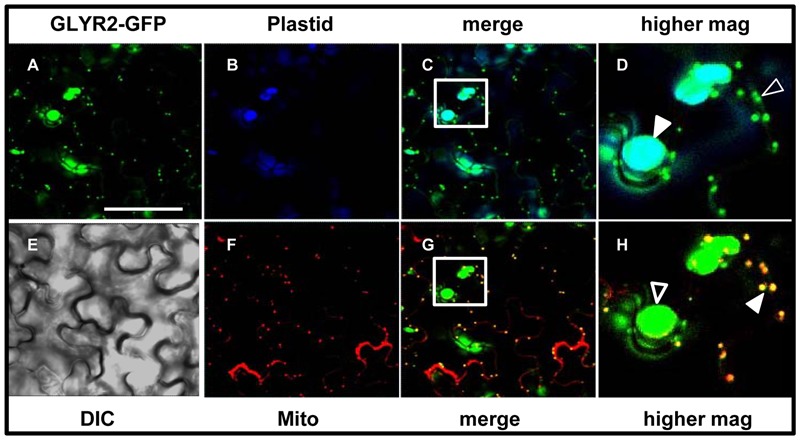
**Dual localization of methoxyfenozide-inducible AtGLYR2-GFP in stably transformed Arabidopsis.** Arabidopsis seedlings, stably expressing *AtGLYR2-GFP* were imaged (by CLSM) after induction with methoxyfenozide **(A)**. **(B,F)** Represent the corresponding chlorophyll autofluorescence (plastid marker) and Mitotracker staining of the same cells, respectively. Co-localization of GFP and chlorophyll is observed based on the cyan color in the merged image in **(C)** and co-localization of GFP and Mitotracker is observed based on the yellow color in the merged image in **(G)**. Boxes correspond to the regions of the cell shown at higher magnification in panels **(D,H)**; solid arrowheads indicate obvious examples of co-localizations, whereas open arrowheads indicate obvious examples of non-co-localization. **(E)** The corresponding differential interference contrast image. Scale bar = 30 μm.

To further eliminate the possibility that the localization of GLYR2 to mitochondria was an artifact of expression in either a non-native system (i.e., tobacco BY-2 cells) or perhaps a distinct cell type (i.e., Arabidopsis suspension cell-derived protoplasts), we generated stably transformed Arabidopsis seedlings expressing methoxyfenozide-inducible *AtGLYR2-GFP*. The presence of the *AtGLYR2-GFP* transgene was confirmed via gene-specific PCR amplification, and the expression of *At*GLYR2, as assessed by quantitative PCR, was observed to be approximately four times higher in transgenic seedlings induced with methoxyfenozide for 48 and 72 h, than in non-treated wild-type (WT) control seedlings or similarly treated WT seedlings, all of which displayed relatively low levels of *AtGLYR2-GFP* (Supplementary Figure S2). Furthermore, allied control experiments revealed that no fluorescence attributable to GFP was observed in WT seedlings in the presence or absence of methoxyfenozide, due to bleed-through from endogenous chlorophyll autofluorescence or Mitotracker staining, or in *At*GLYR2-GFP-transformed seedlings in the absence of methoxyfenozide induction (Supplementary Figure S3). However, upon induction *At*GLYR2-GFP localized consistently to both plastids and mitochondria, based on the co-localization of the fusion protein with chlorophyll autofluorescence and Mitotracker staining, respectively (**Figure 5**), corroborating results for the dual localization of *At*GLYR2-GFP in tobacco BY-2 cells (**Figure 3**) and Arabidopsis protoplasts (**Figure 4**).

Dual targeting of proteins to mitochondria and chloroplasts could be a consequence of alternative gene splicing, transcription and/or translation initiation sites, as well as perhaps an ambiguous targeting signal that is recognized by the import machinery at both organelles (Carrie and Small, 2013). For example, the import of Thr-tRNA synthetase into both mitochondria and chloroplast is considered to involve a shared targeting signal domain and specific organelle receptors (Berglund et al., 2009), whereas the carrier protein Brittle 1 requires distinct targeting information that is recognized by different organelle receptors (Bahaji et al., 2011). In the case of GLYR2, the Arabidopsis Information Resource depicts only one version of the *AtGLYR2* gene and no potential splice variants. Moreover, cloning of *AtGLYR2, MdGLYR2*, or *OsGLYR2* from total cDNA resulted in only one clear sequence corresponding to GLYR2, indicating that GLYR2s in these three species are not alternatively spliced or transcribed, despite the presence of a conserved second methionine 16–18 amino acids downstream of the first N-terminal methionine of *At*GLYR2 and *Md*GLYR2 (Supplemental Figure S1A). With respect to an ambiguous dual targeting signal peptide, while no consensus sequence(s) has been identified to date, N-terminal sequences enriched in positively charged residues (i.e., arginine, histidine and lysine) and deficient in glycine and negatively charged residues (i.e., aspartate and glutamate) (Carrie et al., 2009), or enriched in phenylalanine, leucine and serine and deficient in glycine (Berglund et al., 2009) have been reported. However, the N-terminal sequences of GLYR2s do not generally possess the same amino acid characteristics (Supplementary Figure S1A) and therefore may possess some other yet-to-be identified dual targeting signals, resulting in their localization to both plastids and mitochondria.

### Compartmentation and Potential Function of GLYR Proteins

**FIGURE 6:**
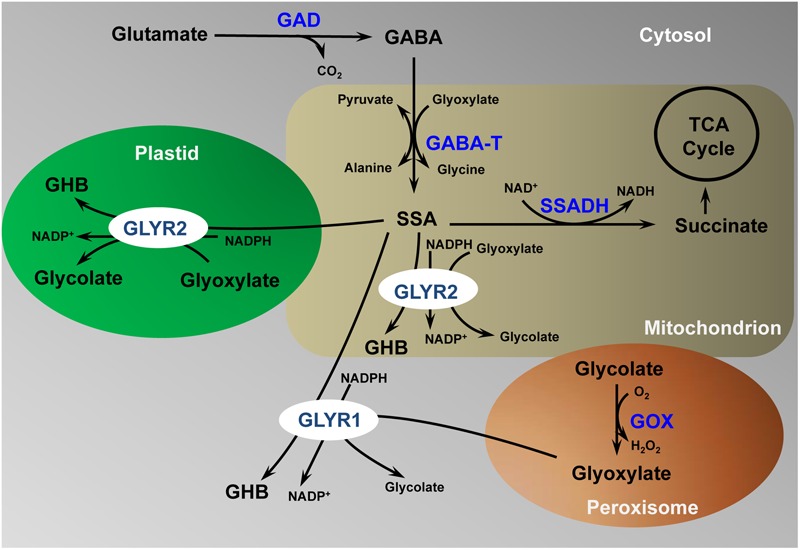
**Proposed model for the detoxification of γ-aminobutyrate-derived succinic semialdehyde and photorespiration-derived glyoxylate in multiple subcellular compartments.** γ-aminobutyrate, GABA; GABA transaminase, GABA-T; glutamate decarboxylase, GAD; glycolate oxidase, GOX; glyoxylate reductase, GLYR; γ-hydroxybutyrate, GHB; succinic semialdehyde, SSA; tri-carboxylic acid cycle, TCA. (Modified from Shelp et al. (2012); permission to reproduce not required)

As depicted in the model in **Figure 6**, cytosolic GLYR1, as well as plastidial and mitochondrial GLYR2, could serve physiological roles in both glyoxylate and SSA metabolism. For example, abiotic stresses such as drought, heat and salinity cause plant stomata to close, leading to reduced intracellular CO_2_/O_2_ ratios and elevated rates of photorespiration, which might result in the accumulation of glycolate and glyoxylate (Allan et al., 2009). Arabidopsis plants exposed to these stresses could have elevated *GLYR* expression, as well as NAD(P)H/NAD(P)^+^ ratios (Allan et al., 2008), suggesting that NADPH-dependent GLYR activity would be stimulated (Allan et al., 2009). In contrast, chilling would be expected to decrease the absolute and relative rates of photorespiration (Foyer et al., 2009), and O_2_ deficiency stresses such as hypoxia and submergence would be expected to suppress the activity of glycolate oxidase (Narsai et al., 2009), so that glyoxylate would not be generated. However, the levels of GABA and *GLYR* expression could increase, together with the NAD(P)H/NAD(P)^+^ ratios, resulting in the diversion of SSA from succinate production to γ-hydroxybutyrate (Allan et al., 2008, 2012). Notably, the submergence-induced accumulation of GHB is decreased in both Arabidopsis *glyr1* and *glyr2* single knockout mutants (Allan et al., 2012). GABA and γ-hydroxybutyrate production has also been observed with many abiotic stresses (Allan et al., 2008). Thus, while the GLYRs are biochemically interchangeable, it can be hypothesized that they function in metabolically diverse cellular compartments.

## Author Contributions

BS conceived the project; BS and GB supervised the project; CB and AZ conducted most of the experiments and data analysis; CT cloned the apple *GLYR* genes; JD, KA, and RM supplied reagents and/or discussed the project; CB, AZ, RM, GB, and BS wrote and/or edited the manuscript; and, all authors read and approved the final manuscript.

## Conflict of Interest Statement

The authors declare that the research was conducted in the absence of any commercial or financial relationships that could be construed as a potential conflict of interest. The research was funded in part by AgroFresh Inc., but they were not involved in experimental design or the decision to publish.

## Footnotes

1 http://blast.ncbi.nlm.nih.gov/Blast.cgi

2 http://www.phytozome.net/

3 https://db.cngb.org/blast4onekp/home

4 www.rosaceae.org

## References

[B1] Allan W. L. Breitkreuz K. E. Waller J. C. Simpson J. P. Hoover G. J. Rochon A. (2012). Detoxification of succinate semialdehyde in *Arabidopsis* glyoxylate reductase and NAD kinase mutants subjected to submergence stress. *Botany* 90 51–61. 10.1139/b11-083

[B2] Allan W. L. Clark S. M. Hoover G. J. Shelp B. J. (2009). Role of plant glyoxylate reductases during stress: a hypothesis. *Biochem. J.* 423 15–22. 10.1042/BJ20090826 19740079 PMC2762691

[B3] Allan W. L. Simpson J. P. Clark S. M. Shelp B. J. (2008). γ-Hydroxybutyrate accumulation in *Arabidopsis* and tobacco plants is a general response to abiotic stress: Putative regulation by redox balance and glyoxylate reductase isoforms. *J. Exp. Bot.* 59 2555–2564. 10.1093/jxb/ern122 18495640 PMC2423657

[B4] Bahaji A. Ovecka M. Barany I. Risueno M. C. Munoz F. J. Baroja-Fernandez E. (2011). Dual targeting to mitochondria and plastids of AtBT1 and ZmBT1, two members of the mitochondrial carrier family. *Plant Cell Physiol.* 52 597–609. 10.1093/pcp/pcr019 21330298

[B5] Bauwe H. Hagemann M. Fernie A. R. (2010). Photorespiration: players, partners and origin. *Trends Plant Sci.* 15 330–336. 10.1016/j.tplants.2010.03.006 20403720

[B6] Becker B. (2013). Snow ball earth and the split of Streptophyta and Chlorophyta. *Trends Plant Sci.* 18 180–183. 10.1016/j.tplants.2012.09.010 23102566

[B7] Berglund A. K. Spånning E. Biverståhl H. Maddalo G. Tellgren-Roth C. Mäler L. (2009). Dual targeting to mitochondria and chloroplasts: characterization of Thr-tRNA synthetase targeting peptide. *Mol. Plant* 2 1298–1309. 10.1093/mp/ssp048 19995731

[B8] Brandizzi F. Irons S. Kearns A. Hawes C. (2003). BY-2 cells: culture and transformation for live cell imaging. *Curr. Protoc. Cell Biol.* 1 1–17. 10.1002/0471143030.cb0107s19 18228413

[B9] Brauer E. K. Rochon A. Bi Y.-M. Bozzo G. B. Rothstein S. J. Shelp B. J. (2011). Reappraisal of nitrogen use efficiency in rice overexpressing *glutamine synthetase 1*. *Physiol. Plant.* 141 361–372. 10.1111/j.1399-3054.2011.01443.x 21214879

[B10] Breitkreuz K. E. Allan W. L. Van Cauwenberghe O. R. Jakobs C. Talibi D. André B. (2003). A novel γ-hydroxybutyrate dehydrogenase: identification and expression of an *Arabidopsis* cDNA and potential role under oxygen deficiency. *J. Biol. Chem.* 278 41552–41556. 10.1074/jbc.M305717200 12882961

[B11] Brocker C. Vasiliou M. Carpenter S. Carpenter C. Zhang Y. Wang X. (2013). Aldehyde dehydrogenase (ALDH) superfamily in plants: gene nomenclature and comparative genomics. *Planta* 237 189–210. 10.1007/s00425-012-1749-0 23007552 PMC3536936

[B12] Carrie C. Giraud E. Whelan J. (2009). Protein transport in organelles: dual targeting of proteins to mitochondria and chloroplasts. *FEBS J.* 276 1187–1195. 10.1111/j.1742-4658.2009.06876.x 19187233

[B13] Carrie C. Small I. (2013). A reevaluation of dual-targeting of proteins to mitochondria and chloroplasts. *Biochim. Biophys. Acta* 1833 253–259. 10.1016/j.bbamcr.2012.05.029 22683762

[B14] Ching S. L. K. Gidda S. K. Rochon A. Van Cauwenberghe O. R. Shelp B. J. Mullen R. T. (2012). Glyoxylate reductase isoform 1 is localized in the cytosol and not peroxisomes in plant cells. *J. Integr. Plant Biol.* 54 152–168. 10.1111/j.1744-7909.2012.01103.x 22309191

[B15] Claros M. G. (1995). MitoProt, a Macintosh application for studying mitochondrial proteins. *Comput. Appl. Biosci.* 11 441–447. 10.1093/bioinformatics/11.4.441 8521054

[B16] Clough S. J. Bent A. F. (1998). Floral dip: a simplified method for *Agrobacterium*-mediated transformation of *Arabidopsis thaliana*. *Plant J.* 16 735–743. 10.1046/j.1365-313x.1998.00343.x 10069079

[B17] Czechowski T. Stitt M. Altmann T. Udvardi M. K. (2005). Genome-wide identification and testing of superior reference genes for transcript normalization. *Plant Physiol.* 139 5–17. 10.1104/pp.105.063743 16166256 PMC1203353

[B18] Denecke J. Aniento F. Frigerio L. Hawes C. Hwang I. Mathur J. (2012). Secretory pathway research: the more experimental systems the better. *Plant Cell* 24 1316–1326. 10.1105/tpc.112.096362 22523202 PMC3398477

[B19] Dietrich C. R. Han G. Chen M. Berg R. H. Dunn T. M. Cahoon E. B. (2008). Loss-of-function mutations and inducible RNAi suppression of Arabidopsis *LCB2* genes reveal the critical role of sphingolipids in gametophytic and sporophytic cell viability. *Plant J.* 54 284–298. 10.1111/j.1365-313X.2008.03420.x 18208516

[B20] Emanuelsson O. Nielsen H. Brunak S. von Heijne G. (2000). Predicting subcellular localization of proteins based on their N-terminal amino acid sequence. *J. Mol. Biol.* 300 1005–1016. 10.1006/jmbi.2000.3903 10891285

[B21] Englert M. Latz A. Becker D. Gimple O. Beier H. Akama K. (2007). Plant pre-tRNA splicing enzymes are targeted to multiple cellular compartments. *Biochimie* 89 1351–1365. 10.1016/j.biochi.2007.06.014 17698277

[B22] Felsenstein J. (1985). Confidence limits on phylogenies: an approach using the bootstrap. *Evolution* 39 783–791. 10.2307/2408678 28561359

[B23] Foyer C. H. Bloom A. J. Queval G. Noctor G. (2009). Photorespiratory metabolism genes, mutants, energetics, and redox signaling. *Annu. Rev. Plant Biol.* 60 455–484. 10.1146/annurev.arplant.043008.091948 19575589

[B24] Frelin O. Agrimi G. Laera V. L. Castegna A. Richardson L. G. L. Mullen R. T. (2012). Identification of mitochondrial thiamin diphosphate carriers from *Arabidopsis* and maize. *Funct. Integr. Genomics* 12 317–326. 10.1007/s10142-012-0273-4 22426856

[B25] Fukasawa Y. Tsuji J. Fu S. C. Tomii K. Horton P. Imai K. (2015). MitoFates: improved prediction of mitochondrial targeting sequences and their cleavage sites. *Mol. Cell. Proteomics* 14 1113–1126. 10.1074/mcp.M114.043083 25670805 PMC4390256

[B26] Gidda S. K. Shockey J. M. Falcone M. Kim P. K. Rothstein S. J. Andrews D. W. (2011). Hydrophobic-domain-dependent protein-protein interactions mediate the localization of GPAT enzymes to ER subdomains. *Traffic* 12 452–472. 10.1111/j.1600-0854.2011.01160.x 21214700

[B27] Givan C. Kleczkowski L. A. (1992). The enzymic reduction of glyoxylate and hydroxypyruvate in leaves of higher plants. *Plant Physiol.* 100 552–556. 10.1104/pp.100.2.552 16653027 PMC1075593

[B28] Givan C. V. Tsutakawa S. Hodgson J. M. David N. Randall D. D. (1988). Glyoxylate reductase activity in pea leaf protoplasts nucleotide specificity and subcellular location. *J. Plant Physiol.* 132 593–599. 10.1016/S0176-1617(88)80260-8

[B29] Hooper C. M. Tanz S. K. Castleden I. R. Vacher M. A. Small I. D. Millar A. H. (2014). SUBAcon: a consensus algorithm for unifying the subcellular localization data of the *Arabidopsis* proteome. *Bioinformatics* 30 3356–3364. 10.1093/bioinformatics/btu550 25150248

[B30] Hoover G. J. Jørgensen R. Rochon A. Bajwa V. S. Merrill A. R. Shelp B. J. (2013). Identification of catalytically important amino acid residues for enzymatic reduction of glyoxylate in plants. *Biochim. Biophys. Acta* 1834 2663–2671. 10.1016/j.bbapap.2013.09.013 24076009

[B31] Hoover G. J. Prentice G. A. Merrill R. A. Shelp B. J. (2007a). Glyoxylate reductase: studies of initial velocity, dead-end inhibition and product inhibition. *Can. J. Bot.* 85 896–902. 10.1139/B07-082

[B32] Hoover G. J. Van Cauwenberghe O. R. Breitkreuz K. E. Clark S. M. Merrill A. R. Shelp B. J. (2007b). Glyoxylate reductase: general biochemical properties and substrate specificity for the recombinant protein, and developmental expression and implications for glyoxylate and succinic semialdehyde metabolism in planta. *Can. J. Bot.* 85 883–895. 10.1139/B07-081

[B33] Horton P. Park K.-J. Obayashi T. Fujita N. Harada H. Adams-Collier C. J. (2007). Wolf PSORT: protein localization predictor. *Nucleic Acids Res.* 35 W585–W587. 10.1093/nar/gkm259 17517783 PMC1933216

[B34] Jones D. T. Taylor W. R. Thornton J. M. (1992). The rapid generation of mutation data matrices from protein sequences. *Comput. Appl. Biosci.* 8 275–282. 10.1093/bioinformatics/8.3.275 1633570

[B35] Kumar S. Stecher G. Tamura K. (2016). MEGA7: molecular evolutionary genetics analysis version 7.0 for bigger datasets. *Mol. Biol. Evol.* 33 1870–1874. 10.1093/molbev/msw054 27004904 PMC8210823

[B36] Millar A. H. Carrie C. Pogson B. Whelan J. (2009). Exploring the function-location nexus: using multiple lines of evidence in defining the subcellular location of plant proteins. *Plant Cell* 21 1625–1631. 10.1105/tpc.109.066019 19561168 PMC2714922

[B37] Narsai R. Howell K. A. Carroll A. Ivanova A. Millar A. H. Whelan J. (2009). Defining core metabolic and transcriptomic responses to oxygen availability in rice embryos and young seedlings. *Plant Physiol.* 151 306–322. 10.1104/pp.109.142026 19571305 PMC2736006

[B38] Park J. Khuu N. Howard A. S. Mullen R. T. Plaxton W. C. (2012). Bacterial- and plant-type phosphoenolpyruvate carboxylase isozymes from developing castor oil seeds interact *in vivo* and associate with the surface of mitochondria. *Plant J.* 71 251–262. 10.1111/j.1365-313X.2012.04985.x 22404138

[B39] Reumann S. Chowdhary G. Lingner T. (2016). Characterization, prediction and evolution of plant targeting type 1 (PTS1s). *Biochim. Biophys. Acta* 1863 790–803. 10.1016/j.bbamcr.2016.01.001 26772785

[B40] Saravanan V. Lakshmi P. T. V. (2014). Dualpred: a webserver for predicting plant proteins dual-targeted to chloroplast and mitochondria using split protein-relatedness-measure feature. *Curr. Bioinform.* 10 323–331. 10.2174/1574893609666140226000041

[B41] Schirawski J. Planchais S. Haenni A. L. (2000). An improved protocol for the preparation of protoplasts from an established *Arabidopsis thaliana* cell suspension culture and infection with RNA of turnip yellow mosaic tymovirus: a simple and reliable method. *J. Virol. Methods* 86 85–94. 10.1016/S0166-0934(99)00173-1 10713379

[B42] Shaner N. C. Campbell R. E. Steinbach P. A. Giepmans B. N. Palmer A. E. Tsien R. Y. (2004). Improved monomeric red, orange and yellow fluorescent proteins derived from *Discosoma* sp. red fluorescent protein. *Nat. Biotechnol.* 22 1567–1572. 10.1038/nbt1037 15558047

[B43] Shelp B. J. Mullen R. T. Waller J. C. (2012). Compartmentation of GABA metabolism raises intriguing questions. *Trends Plant Sci.* 17 57–59. 10.1016/j.tplants.2011.12.006 22226724

[B44] Simpson J. P. Di Leo R. Dhanoa P. K. Allan W. L. Makhmoudova A. Clark S. M. (2008). Identification and characterization of a plastid-localized *Arabidopsis* glyoxylate reductase isoform: comparison with a cytosolic isoform and implications for cellular redox homeostasis and aldehyde detoxification. *J. Exp. Bot.* 59 2545–2554. 10.1093/jxb/ern123 18495639 PMC2423656

[B45] Trobacher C. P. Clark S. M. Bozzo G. G. Mullen R. T. DeEll J. R. Shelp B. J. (2013a). Catabolism of GABA in apple fruit: subcellular localization and biochemical characterization of two γ-aminobutyrate transaminases. *Postharv. Biol. Technol.* 75 106–113. 10.1016/j.postharvbio.2012.08.005

[B46] Trobacher C. P. Zarei A. Liu J. Clark S. M. Bozzo G. G. Shelp B. J. (2013b). Calmodulin-dependent and calmodulin-independent glutamate decarboxylases in apple fruit. *BMC Plant Biol.* 13:144 10.1186/1471-2229-13-144 PMC3849887 24074460

[B47] Zarei A. Körbes A. P. Younessi P. Montiel G. Champion A. Memelink J. (2011). Two GCC boxes and AP2/ERF-domain transcription factor ORA59 in jasmonate/ethylene-mediated activation of the *PDF1.2* promoter in Arabidopsis. *Plant Mol. Biol.* 75 321–331. 10.1007/s11103-010-9728-y 21246258 PMC3044237

[B48] Zarei A. Trobacher C. P. Cooke A. R. Meyers A. J. Hall J. C. Shelp B. J. (2014). Apple fruit copper amine oxidase isoforms: peroxisomal MdAO1 prefers diamines as substrates, whereas extracellular MdAO2 exclusively utilizes monoamines. *Plant Cell Physiol.* 56 137–147. 10.1093/pcp/pcu155 25378687

[B49] Zelitch I. (1953). Oxidation and reduction of glycolic and glyoxylic acids in plants. II. Glyoxylic acid reductases. *J. Biol. Chem.* 201 719–726. 13061410

[B50] Zhang Y. Zheng Y. Qin L. Wang S. Buchko G. W. (2014). Structural characterization of a β-hydroxyacid dehydrogenase from *Geobacter sulfurreducens* and *Geobacter metallireducens* with succinic semialdehyde activity. *Biochimie* 104 61–69. 10.1016/j.biochi.2014.05.002 24878278

