# Supplementary material for: Ancient Plant Glyoxylate/Succinic Semialdehyde Reductases: GLYR1s Are Cytosolic, Whereas GLYR2s Are Localized to Both Mitochondria and Plastids
Source: Front Plant Sci. 2017 Apr 21;8:601. doi: 10.3389/fpls.2017.00601 (PMC5399074; doi:10.3389/fpls.2017.00601)
Supplement: Supplementary file 1 [file Table_1.PDF]

SUPPLEMENTARY DATA FILES

Ancient Plant Glyoxylate/Succinic Semialdehyde Reductases: GLYR1s are Cytosolic, whereas GLYR2s are Localized to Both Mitochondria and Plastids

Carolyne J. Brikis[1], Adel Zarei[1], Christopher P. Trobacher[1], Jennifer R. DeEll[2], Kazuhito Akama[3], Robert T. Mullen[4], Gale G. Bozzo[1], and Barry J. Shelp[1]

[1]Department of Plant Agriculture, University of Guelph, Guelph, Ontario, Canada N1G 2W1. [2]Ontario Ministry of Agriculture Food and Rural Affairs, Box 587, 1283 Blueline Rd. at Highway 3, Simcoe, ON N3Y 4N5, Canada. [3]Department of Biological Science, Shimane University, 1060 Nishikawatsu-chou, Matsue 690-8504, Japan. [4]Department of Molecular and Cellular Biology, University of Guelph, Guelph, Ontario, Canada N1G 2W1.

SUPPLEMENTARY FIGURES

A. Multiple sequence alignment for GLYRs

```
AtGLYR1         ------------------------------------------------------------
MdGLYR1         ------------------------------------------------------------
OsGLYR1         ------------------------------------------------------------
AtGLYR2         MPLVSLSFASS--SSKAMALCS-ICPRIPLRFRPKPISPFLSKPQICLAYRVYSSLQSTT 57
MdGLYR2         MSLLVKTTNCSRLSSTAMAVCSSFCPHIPTRLT----KSFPAKP-HSLSFKAFSS-QASN 54
OsGLYR2         --------------MAAMAAASLLCARAAAAAP-----TLRLRGGGRGARLVFSCSASSS 41

AtGLYR1         ---------MEVGFLGLGIMGKAMSMNLLKNGFKVTVWNRTLSKCDELVEHGASVCESPA 51
MdGLYR1         ---------MEVGFLGLGIMGKAMSMNLLRHGFKVTVWNRTLSKSHELVEHGASVAETPA 51
OsGLYR1         ---------MEVGFLGLGIMGKAMAANLLRHGFRVTVWNRTLSKCQELVALGAAVGETPA 51
AtGLYR2         PSTRDELGTVSIGFLGMGIMGSPMAQNLIKAGCDVTVWNRTKSKCDPLVGLGAKYKSSPE 117
MdGLYR2         ASSKDEL-PARVGFLGLGIMGSPMAQNLIKSGCDVTVWNRTKSKCDPLISLGAKYKPSPE 113
OsGLYR2         SPSGEGGFSGKVGFLGLGIMGAPMASNLINAGCDVTVWNRTRSKCDPLLSLGAKYEPSPA 101
                           :****:**** .*: **:.  *  ******* **.. *:  **    :*

AtGLYR1         EVIKKCKYTIAMLSDPCAALSVVFDKGGVLEQICEGKGYIDMSTVDAETSLKINEAITGK 111
MdGLYR1         EVVRKCKYTIAMLSDPSAALSVVFGKDGILEQICAGKSYVDMSTVDADTSSKINEAIKER 111
OsGLYR1         AVVAKCRYTIAMLSDPSAALSVVFDKDGVLEQIGEGKGYVDMSTVDAATSCKISEAIKQK 111
AtGLYR2         EVTATCDLTFAMLADPESAIDVACGKNGAIFGISSGKGYVDVSTVDVASSILISKQIKDT 177
MdGLYR2         EVAASCDVTFAMLADPESAVAVALGKHGAANGMSSGKGYVDVSTVDVATSKLIGGNIKAT 173
OsGLYR2         DVASSCDVTFAMLADPESAVEVACGANGAAQGMAPGKGYVDVSTVDAATSKLIGKHITST 161
                 *  .*  *:***:** :*: *. .  *    :  **.*:*:****. :*  *.  *.

AtGLYR1         GGRFVEGPVSGSKKPAEDGQLIILAAGDKALFEESIPAFDVLGKRSFYLGQVGNGAKMKL 171
MdGLYR1         GGYFLEAPVSGSKKPAEDGQLVILAAGEKALYEEVIPAFNVMGKKSFYLGQVGNGAKMKL 171
OsGLYR1         GGAFVEAPVSGSKKPAEDGQLVILAAGDKVLYDDMVPAFDVLGKKSFFLGEIGNGAKMKL 171
AtGLYR2         GALFLEAPVSGSKKPAEDGQLIFLTAGDKPLYEKAAPFLDIMGKSKFYLGEVGNGAAMKL 237
MdGLYR2         GASFLEAPVSGSKKPAEDGQLIFLTAGDKSLYETVASLLDIMGKSRFYLGEVGNGAAMKL 233
OsGLYR2         GASFLEAPVSGSKKPAEDGLLIFLTAGDESLYNRVASLLDVMGKSRFFLGDVGKGADMKL 221
                *. *:*.************ *::*:**:: *::   .  ::::**  *:**::*:** ***

AtGLYR1         IVNMIMGSMMNAFSEGLVLADKSGLSSDTLLDILDLGAMTNPMFKGKGPSMNKSSYPPAF 231
MdGLYR1         VVNMIMGSMMNAFSEGLVLAGRSGLEPSVLLDVLDLGGIANPMFRLKGPTMIQGSHSPAF 231
OsGLYR1         VVNMIMGSMMNALSEGLSLADNSGLSPQTLLDVLDLGAIANPMFKLKGPSMLQGSYNPAF 231
AtGLYR2         VVNMIMGSMMASFAEGILLSQKVGLDPNVLVEVVSQGAINAPMYSLKGPSMIKSVYPTAF 297
MdGLYR2         VVNMIMGSMMASFSEGLLLTEKIGLDPKVLVEVVSQGAISAPMYSMKGPSMIQSVYPTAF 293
OsGLYR2         VVNMVMGSMMVSFSEGLLLSEKVGLDPNTLVEVISQGAISAPMFSLKGPSMVKAAYPTAF 281
                :***:***** :::**: *: . **....*::::. *.:  **:  ***:* :. : .**

AtGLYR1         PLKHQQKDMRLALALGDENAVSMPVAAAANEAFKKARSLGLGDLDFSAVIEAVKFSRE-- 289
MdGLYR1         PLKHQQKDMRLALALGDETATSMPVAAAANEAFKKARSMGLGDLDFSAVYETVKTLEEPS 291
OsGLYR1         PLKHQQKDMRLALALGDENAVSMPVAAASNEAFKKARSLGLGDLDFSAVYEVLKGAGGSG 291
AtGLYR2         PLKHQQKDMRLALGLAESVSQSTPIAAAANELYKVAKSYGLSDEDFSAVIEALKAAKSRE 357
MdGLYR2         PLKHQQKDMRLALGLAESVSQSTPIAAAANELYKVAKSHGLSDEDFSAVIEALKPKLKH- 352
OsGLYR2         PLKHQQKDLRLALALAESVSQSIPTVAAANELYKVAKSLGLADQDFSAVIEALKAKEQSK 341
                ********:****.*.:. : * * .**:** :* *:* **.* ***** *.:*

AtGLYR1         ------
MdGLYR1         RNSQLR 297
OsGLYR1         KA---- 293
AtGLYR2         A----- 358
MdGLYR2         ------
OsGLYR2         ------
```

Identity matrix comparisons of GLYRs

	AtGLYR1	MdGLYR1	OsGLYR1	AtGLYR2	MdGLYR2	OsGLYR2
1) AtGLYR1	100.00	80.28	79.58	57.44	57.79	56.75
2) MdGLYR1	80.28	100.00	78.84	55.82	57.24	54.64
3) OsGLYR1	79.58	78.84	100.00	57.19	57.59	57.04
4) AtGLYR2	57.44	55.82	57.19	100.00	77.36	67.06
5) MdGLYR2	57.79	57.24	57.59	77.36	100.00	71.51
6) OsGLYR2	56.75	54.64	57.04	67.06	71.51	100.00

B. Predicted subcellular localization of GLYR2s

	*At*GLYR2		*Md*GLYR2		*Os*GLYR2	
Program	Chl[a]	Mit[b]	Chl	Mit	Chl	Mit
TargetP	0.819	0.108	0.662	0.212	0.590	0.560
WoLF PSORT	10.0	4.0	11.0	2.0	13.0	---
Plant PLoc	Yes	No	Yes	No	Yes	No
AtSubP	-1.14	1.14	---	---	---	---
MitoProt	---	0.99	---	0.98	---	0.87
Chloro P	Yes	---	Yes	---	Yes	---
MitoFates	---	0.02	---	0.04	---	0.42
MultiLoc/TargetLoc	0.98	0.01	0.07	0.92	0.3	0.7
DualPred	Yes	No	No	Yes	No	Yes
SUBA3	Yes	No	---	---	---	---

[a]Chl, chloroplast;
[b]Mit, mitochondria

Supplementary Figure S1. Multiple sequence alignment, percent identity matrix and predicted subcellular localization of apple (*Malus* x *domestica*) GLYR1 (GenBank Acc. No. KT202799) and GLYR2 (KT2027800), rice (*Oryza sativa*) GLYR1 (XM-015767691) and GLYR2 (AK064876), and Arabidopsis (*Arabidopsis thaliana*) GLYR1 (NM 113449) and GLYR2 (NM 101628) (A). The alignment and percent identity matrix was created with ClustalW2 (EMBL-EBI). Identical residues are marked with an asterisk, and similar and non-similar residues are

marked with a colon and period, respectively. Active site residues are highlighted in grey and predicted N-terminal targeting sequences are underlined. The blue bar denotes the glycine-rich fingerprint motif and conserved glycines are highlighted in yellow. (B). *In silico* analysis of subcellular localization was conducted using various websites (http://www.cbs.dtu.dk/services/TargetP/; http://www.genscript.com/psort/wolf_psort.html; http://www.csbio.sjtu.edu.cn/bioinf/plant/; http://bioinfo3.noble.org/AtSubP/?dowhat=About; http://ihg.gsf.de/ihg/mitoprot.html; http://www.cbs.dtu.dk/services/ChloroP/; http://mitf.cbrc.jp/MitoFates/cgi-bin/top.cgi/; http://abi.inf.uni-tuebingen.de/Services/MultiLoc/; http://suba3.plantenergy.uwa.edu.au/; http://pcmpred.bicpu.edu.in/pcmpred.php). A higher or more positive number in each row of the subcellular localization table indicates higher probability of the predicted localization. Dashed lines indicate lack of predictions.

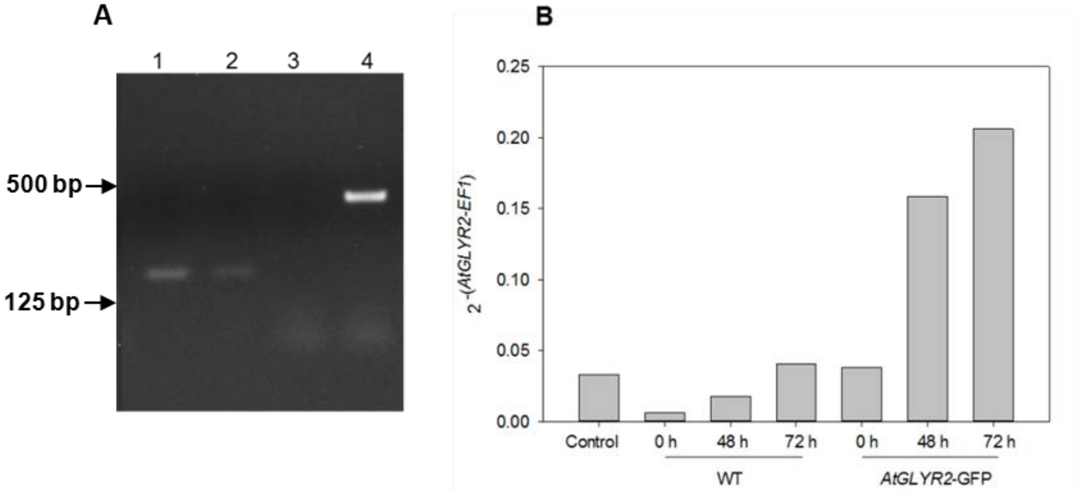

Supplementary Figure S2. Characterization of a stable *AtGLYR2-GFP* transgenic line. (A) Amplification of the *AtGLYR2-GFP* transgene from Arabidopsis genomic DNA. Lanes 1 and 2: amplification of *AtGABA-T* from WT and *AtGLYR2-GFP* transgenic mutant, respectively, resulting in two 161 bp amplicons using RTAtGABA-T-F and RTAtGABA-T-R (Supplementary Table S3). Lane 3: amplification of *AtGLYR2*-GFP from WT with pEC291-F and pEC291-R primers flanking the *AtGLYR2*-GFP fusion, resulting in no amplicon. Lane 4: amplification of *AtGLYR2-GFP* from transgenic *AtGLYR2-GFP*, resulting in a 384 bp amplicon. PCR product form lane 4 was sequenced, and matched the predicted transgene sequence. (B) Expression of *AtGLYR2* from cDNA of WT and *AtGLYR2-GFP* at 0 h, 48 h and 72 h after induction with methoxyfenozide, with RTGLYR2-F and RTGLYR2-F primers as measured by quantitative real time PCR using the house keeping gene *ELONGATION FACTOR-1 ALPHA* (*EF-1*). Control: RNA treated by DNAseI without reverse transcriptase in cDNA reaction.

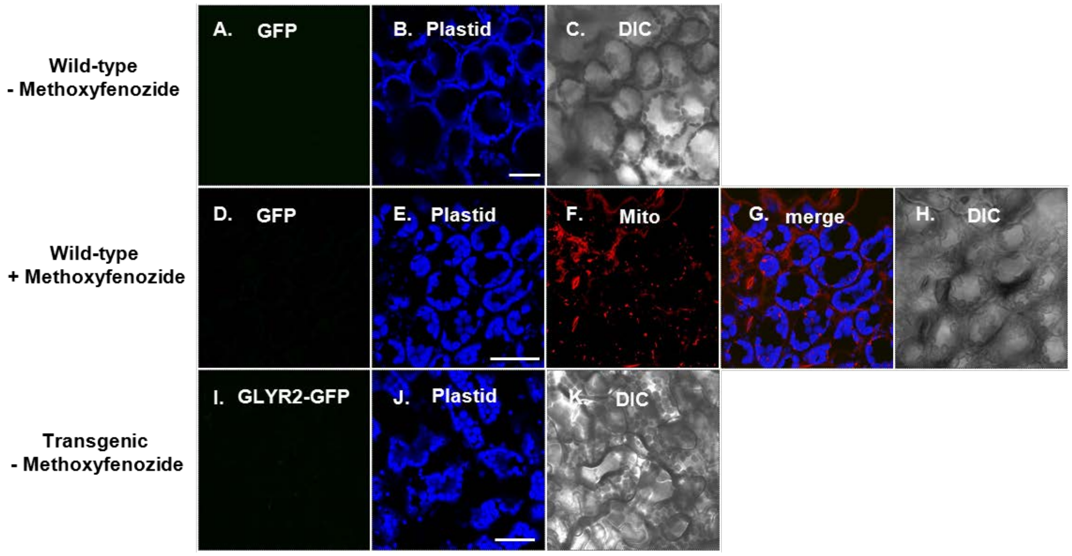

Supplementary Figure S3. Allied controls for inducible *AtGLYR2-GFP* expression in stably-transformed Arabidopsis. Arabidopsis WT seedlings were imaged (by CLSM) without and with methoxyfenozide induction (A and D, respectively). Stably-inducible *AtGLYR2-GFP* also did not display any detectable GFP signal without methoxyfenozide (I). Second column from the left represents the chlorophyll autofluorescence (plastid marker) in WT (B and E) and *AtGLYR2-GFP* lines (J). F represents the Mitotracker staining of the same cells and did not display bleed-through (D) or colocalize with chlorophyll autofluorscence (G). C, H, K are the corresponding differential interference contrast images. Scale bar = 30 µm. fluorescence.

SUPPLEMENTARY TABLES

Supplementary Table S1. List of Reference Sequence ID numbers for plant GLYRs used in construction of the phylogenetic tree.

Species	GLYR1	GLYR2
Arabidopsis thaliana	NP_566768.1	NP_564030.2
Beta vulgaris	XP_010679043	XP_010685374
Brassica rapa	XP_009102475	XP_009149217
Camelina sativa	NP_010488656	NP_010459371
Capsicum annuum	XP_016541146	XP_016562555
Capcella rubella	XP_006298016	XP_006303633
Chlamydomonas reinhardtii	XP_001698820	Cre06g278148
Citrus sinensis [α]	XP_006469385	XP_006466034
Cucumis sativus [α]	----	XP_004146816, XP_994146817
Cucumis melo	----	XP_008447632, XP_008447637
Daucus carota	XP_017258257	XP_017232049
Glycine max	XP_003548573	XP_003543588
Gossypium hirsutum	XP_016744950	XP_016678349
Klebsormidium flaccidum	GAQ83046	GAQ92564
Malus domestica	XP_008391671	XP_008391335
Medicago trunculata	XP_013444705	XP_003597590
Nicotiana sylverstris	XP_009766090	XP_009792478
Nicotiana tubacum	XP_016473866	XP_016459872
Oryza sativa	XP_015623177	XP_015615472
Pinus radiata [β]	DZQM-2053498	DZQM-2013950
Phoenix dactylifera	XP_008801504	XP_008807948
Prunus persica	XP_007202393	XP_007215647
Populus euphratica	XP_011014789	XP_011008201
Physcomitrella patens	XP_001784857	XP_001753791
Solanum lycopersicum	NP_001233832	NP_001233836
Sorghum bicolor	XP_002452295	XP_002458034
Taxus bacata [β]	WWSS-2043489	WWSS-2010652
Theobrama cacao	XP_007049486	XP_0070224477
Vitis vinifera	XP_002266252	XP_002280297
Volvox cateri	XP_002954857	XP_002946110
Zea mays	NP_001148591	XP_002458034

[α] *Cucumis sativus* and *Cucumis melo* ID numbers represent GLYR2A and GLYR2B.

[β] GLYR1 and GLYR2 are predicted proteins from nucleotide scaffold (https://www.bioinfodata.org/Blast4OneKP/blast).

Supplementary Table S2. List of Reference Sequence ID numbers for GLYRs from primitive plants possessing only a single GLYR and used in construction of the phylogenetic tree.

Species	GLYR
Chlorella varibillis	XP_005851967
Coccomyxa subellipsoidea	XP_005648885
Micromonas pusilla	XP_003055572
Ostreococcus lucimarinus	XP_001417895

Supplementary Table S3. Synthetic oligonucleotides

Primer name	Sequence (5' – 3')
CB-F1	CGCCATGGAGGTCGGGTTTCTGGGGCT
CB-R1	GCCCATGGCACGCAGTTGGCTGTTTCG
CB-F2	GCGCTAGCATGTCCTTGTTGGTAAAGACCACC
CB-R2	GGGCTAGCGTGCTTCAACTTCGGTTTCAATG
NheI-AtGLYR2-F	CGGCTAGCATGCCTTTGGTTTCATTATCTTTTGC
NheI-AtGLYR2-R	TGCAAAATCCCGAGAAGCTGCTAGCGC
F-PacI-LAtGR2-GFP	GCTTAATTAACATGCCTTTGGTTTCATTATCTTTT
R-SpeI-LAtGR2-GFP	GCATGGACGAGCTGTACAAGTAAACTAGTCG
RTAtGABA-T-F	CAATAAATCTCCGAACGAACCAT
RTAtGABA-T-R	TCCAAAGAATGCGCCAACA
pEC291-F	TTAAAGCATCAGCAGAAGGATATGAG
pEC291-R	GTGAAGTGGTCACGAGGGA
RTGLYR2-F	GAGCAAATGTGATCCTCTCG
RTGLYR2-R	CAAGCATTGCAAATGTGAGA
RTEF-1-F	TGACAGGCGTTCTGGTAAGGA
RTEF-1-R	CCAGCGTCACCATTCTTCAA
*Not*I-OsGLYR2-F	TAAGCGGCCGCATGGCGGCGATGGCGGCG
*Xho*I-OsGLYR2-R	TTTTCTCGAGCTTGCTCTGCTCCTTTGCCT
